# A role for the Tec family kinase ITK in regulating SEB induced Interleukin-2 production *in vivo *via c-jun phosphorylation

**DOI:** 10.1186/1471-2172-6-19

**Published:** 2005-07-22

**Authors:** Melanie J Ragin, Jianfang Hu, Andrew J Henderson, Avery August

**Affiliations:** 1 Pathobiology Graduate Program, Center for Molecular Immunology & Infectious Disease and Department of Veterinary Science, The Pennsylvania State University, University Park, PA 16802, USA; 2 Immunobiology Option of the Integrated Bioscience Graduate Program, Center for Molecular Immunology & Infectious Disease and Department of Veterinary Science, The Pennsylvania State University, University Park, PA 16802, USA; 3 Center for Molecular Immunology & Infectious Disease and Department of Veterinary Science The Pennsylvania State University, University Park, PA 16802, USA

## Abstract

**Background:**

Exposure to Staphylococcal Enterotoxin B (SEB), a bacterial superantigen secreted by the Gram-positive bacteria *Staphyloccocus aureus*, results in the expansion and eventual clonal deletion and anergy of Vβ8^+ ^T cells, as well as massive cytokine release, including Interleukin-2 (IL-2). This IL-2 is rapidly secreted following exposure to SEB and may contribute to the symptoms seen following exposure to this bacterial toxin. The Tec family kinase ITK has been shown to be important for the production of IL-2 by T cells stimulated *in vitro *and may represent a good target for blocking the production of this cytokine *in vivo*. In order to determine if ITK represents such a target, mice lacking ITK were analyzed for their response to SEB exposure.

**Results:**

It was found that T cells from mice lacking ITK exhibited significantly reduced proliferative responses to SEB exposure *in vitro*, as well as *in vivo*. Examination of IL-2 production revealed that ITK null mice produced reduced levels of this cytokine *in vitro*, and more dramatically, *in vivo*. *In vivo *analysis of c-jun phosphorylation, previously shown to be critical for regulating IL-2 production, revealed that this pathway was specifically activated in SEB reactive Vβ8^+ ^(but not non-reactive Vβ6^+^) T cells from WT mice, but not in Vβ8^+ ^T cells from ITK null mice. However, toxicity analysis indicated that both WT and ITK null animals were similarly affected by SEB exposure.

**Conclusion:**

These data show that ITK is required for IL-2 production induced by SEB *in vivo*, and may regulate signals leading IL-2 production, in part by regulating phosphorylation of c-jun. The data also suggest that perturbing T cell activation pathways leading to IL-2 does not necessarily lead to improved responses to SEB toxicity.

## Background

Superantigens (SAGs) are microbial toxins of bacterial and viral origin with the ability to activate 5–20% of the T cell population, causing T cell activation, cytokine release and systemic shock [1,2]. Most SAGs share the ability to simultaneously bind the class II major histocompatibility complex molecules and the variable region of the T cell receptor β-chain, without the need to be processed by antigen presenting cells [1,2]. Thus SEB can interact directly with MHC class II molecules on APCs and activate T cells bearing the proper TcR Vβ chains. The result of this interaction is large-scale stimulation of any T cell that expresses the proper TCR Vβ chain. A number of studies have shown that when mice are challenged with a SAG such as Staphylococcal Enterotoxin B (SEB), toxicity results from massive induction of cytokines derived from T-helper-type-1 (T_H_1) type cells such as IL-2, IFN-γ, TNF-α and TNF-β [1,3]. This cytokine production is accompanied by expansion of the numbers of SEB reactive T cells, followed by cell death and the induction of functional "anergy" [4-6].

In order for a T cell to be activated antigen presenting cells (APC) must present antigen, either an antigenic peptide or SAG. *In vitro*, this interaction with the TcR has been shown to lead to the activation of a number of tyrosine kinases, including the Src family kinase Lck, the Syk family kinase Zap-70 and the Tec family kinase ITK (for review see [7-10]). This then results in the activation of a number of signaling pathways including members of the MAPK family of kinases, ERK, JNK and p38, followed by transcription factor activation [10]. In vivo, activation of these T cells lead to the induction of cytokine secretion within hours of SEB exposure [1,3].

ITK is expressed primarily in T cells, NK cells and mast cells [11-13]. In T cells, it is rapidly activated following TcR crosslinking *in vitro *[14,15]. Mice lacking ITK exhibit reduced proliferation and IL-2 production *in vitro*, and reduced T cell differentiation *in vitro *and *in vivo*, with T_H_2 cell differentiation preferentially affected [15-19]. The observed reduced proliferation of ITK null T cells *in vitro *is IL-2 dependent as it could be rescued by the addition of exogenous IL-2 [20]. However, these animals are not entirely immunocompromised, with residual responses against LCM, Vaccinia and VS viruses [21]. We have tested whether ITK null mice are susceptible to SEB induced IL-2 secretion. We show here that mice lacking ITK have much reduced IL-2 production and T cell expansion in response to SEB *in vitro *and *in vivo*. We also show that SEB induced the activation of the JNK MAPK pathway in responding T cells *in vivo*, and that ITK null T cells were defective in the activation of this pathway *in vivo*. However, toxicity analysis indicated that both WT and ITK null animals were similarly affected by SEB exposure. Our data suggest that ITK is required for full IL-2 secretion following SEB exposure, and that this may be due to the regulation of the JNK pathway by ITK *in vivo*. However, reducing T cell signals does not necessarily lead to better physiological responses to SEB exposure.

## Results

### ITK deficient T cells proliferate less efficiently than WT T cells in response to varying concentrations of SEB *in vitro*

**Figure 1:**
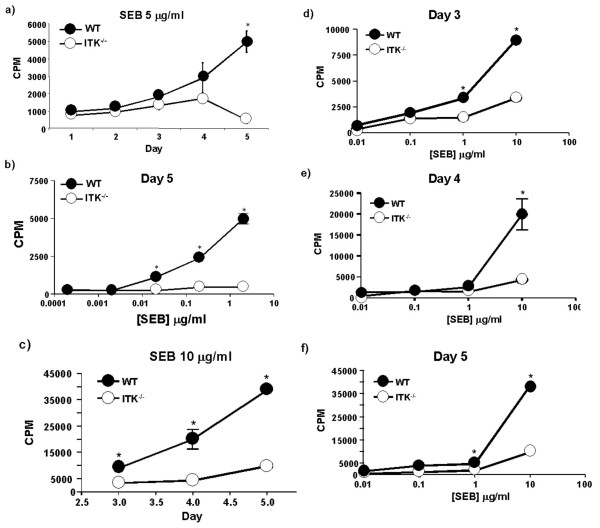
**ITK regulates proliferation *in vitro *in response to SEB**. Splenocytes and lymph node cells from WT (filled circles) and ITK deficient (open circles) mice were stimulated in vitro with the indicated concentrations of SEB, and proliferative response determined. Time course of proliferation over the indicated doses of SEB **a) **5 μg/ml SEB, **b) **dose curve of 0.0005, 0.005, 0.05, 0.5, & 5 μg/ml SEB analyzed at 5 days post stimulation. Purified WT (filled circles) and ITK deficient (open circles) T cells were stimulated in vitro over the indicated time with **c) **10 μg/ml SEB, or a dose curve of 0.01, 0.1, 1, and 10 μg/ml SEB and analyzed at **d) **3 days, **e) **4 days or **f) **5 days post stimulation. * indicates statistically significant difference with a p < 0.05.

It has previously been reported that T cells from mice lacking ITK exhibit much reduced proliferation in response to anti-CD3 or TcR antibodies *in vitro *[15,16,18]. By contrast, these T cells did not have any defects and actually had higher proliferative responses following anti-CD28/PMA stimulation [20]. These data do not provide clear answers as to whether ITK would present a good target for inhibition of SEB induced T cell activation *in vivo*, and in particular, in the cytokine response observed following SAG exposure. We therefore first tested whether ITK null T cells could respond to SEB stimulation by proliferation *in vitro*. Pooled splenocytes and lymph node cells from WT or ITK null animals were cultured *in vitro *in the presence of varying concentrations of SEB (5 μg/ml, 0.5 μg/ml, 0.05 μg/ml, 0.005 μg/ml, and 0.0005 μg/ml), and proliferation analyzed over a 5-day period. We found that WT cells responded normally by proliferating between days 2 and 3, with maximum proliferation observed at day 5 and the 5 μg/ml dose of SEB (Fig. 1a). Analysis of the dose response at 5 days indicated that the WT cells responded in a dose dependent fashion (Fig. 1b). By contrast, cells from ITK mice showed very little response, regardless of the dose of SEB used for stimulation (Fig. 1a, b). We next determined if the same results would be obtained in purified T cell populations from these mice. T cells were purified from WT and ITK null mice, and stimulated with varying dose of SEB, in the presence of APCs. Analysis of the data over the 5 days indicated that in the presence of the highest concentration of SEB tested (10 μg/ml), WT T cells responded more robustly than ITK null T cells (see Fig. 1c). Indeed, at all the doses tested, our analysis indicated that ITK null T cells responded less than WT T cells on day 3, 4 and 5 (Fig. 1d–f), while proliferation was equivalent when PMA and Ionomycin was used to bypass TcR signals (data not shown). These data indicate that ITK null T cells have significantly reduced proliferative responses to SEB *in vitro*.

### ITK null T cells secrete less IL-2 in response to SEB *in vitro*

**Figure 2:**
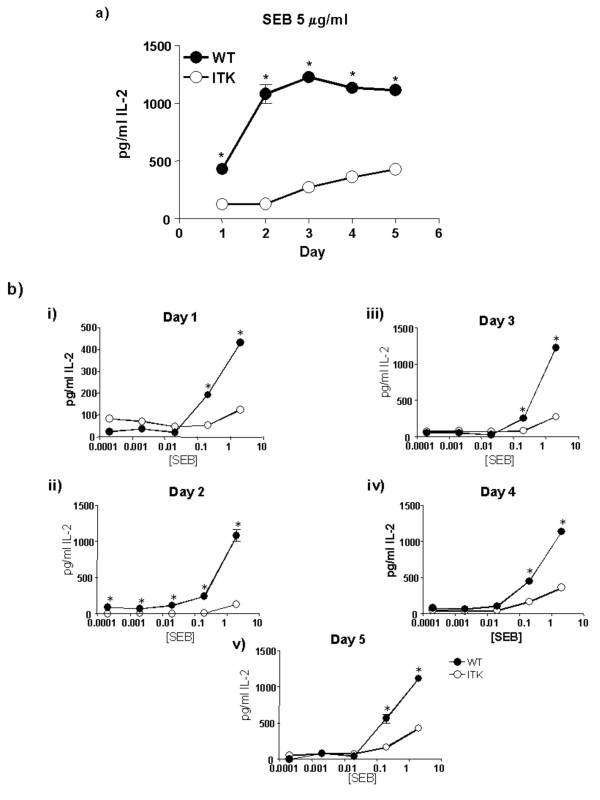
**ITK regulates IL-2 secretion *in vitro *in response to SEB**. Splenocytes and lymph node cells from WT (filled circles) or ITK deficient (open circles) mice were stimulated in vitro with the indicated concentrations of SEB and IL-2 levels secreted in supernatants determined. **a) **Time course of IL-2 secretion at 5 μg/ml SEB dose. **b) **IL-2 secretion over the indicated times and doses. **(i) **Day 1; **(ii) **Day 2; **(iii) **Day 3; **(iv) **Day 4 and **(v) **Day 5. * indicates statistically significant difference with a p < 0.05.

Interleukin-2 is one of the cytokines induced by exposure to SEB [22]. ITK has been shown to regulate IL-2 secretion *in vitro *by T cells stimulated anti-CD3/TcR antibodies or in response to peptide antigen [15,18,23,24]. We therefore determined the levels of IL-2 in supernatants from cells similarly stimulated with SEB *in vitro *(Fig. 2). While WT T cells responded to SEB by secretion of IL-2 within 24 hrs. of stimulation in a dose dependent manner (see Fig. 2a for 5 μg/ml dose), starting at the 0.5 μg/ml SEB concentration (Fig. 2bi), ITK null T cells made very little IL-2, and the small amount of IL-2 that was made was only at the highest concentration of SEB tested (5 μg/ml). ITK null T cells consistently secreted less IL-2 over the 5-day period and dose range (Fig. 2b). These data indicate that as previously reported for TcR or antigenic stimulation, ITK regulates the production of IL-2 in vitro in response to SEB stimulation [15,18,23,24].

### Reduced expansion of Vβ8^+^CD4^+ ^population in ITK null animals in response to SEB exposure *in vivo*

**Figure 3:**
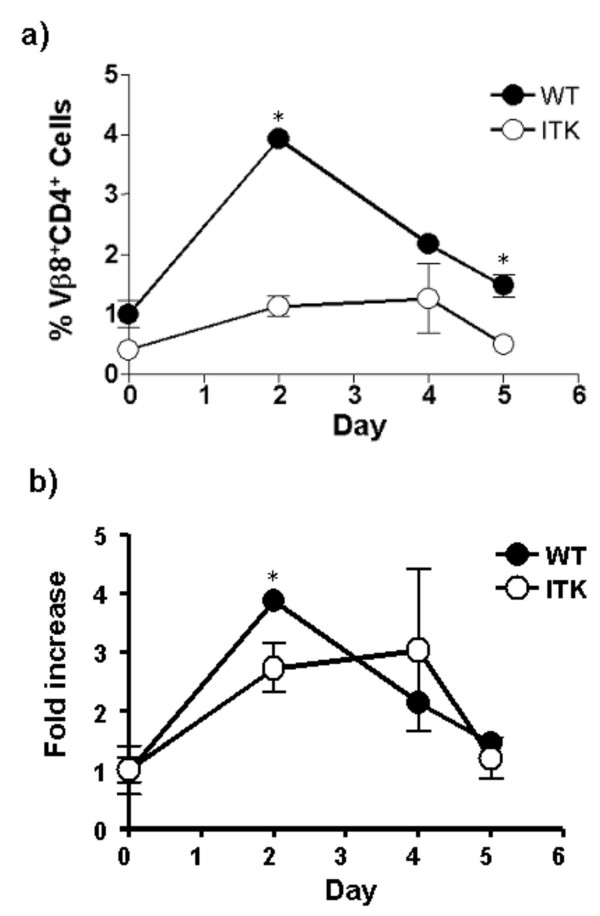
**Reduced T cell expansion in response to SEB exposure *in vivo***. **a) **WT (filled circles) or ITK deficient mice (open circles) were injected with 50 μg SEB i.p. and peripheral blood lymphocytes analyzed after 2, 4 and 5 days as in the materials and methods for the percentage of Vβ8^+^CD4^+ ^T cells as indicated (Vβ6^+^CD4^+ ^T cells showed no change, data not shown). **b) **The same data plotted as fold increase in percentage of Vβ8^+^CD4^+ ^T cells in peripheral blood. * indicates a statistically significant difference with a p < 0.05.

While these data indicate that ITK regulates T cell proliferation in response to SEB *in vitro*, it is possible that within the context of antigen presentation and optimal co-stimulatory signals *in vivo*, ITK null T cells may exhibit a better response. Exposure of animals to SEB results in an expansion of the SEB reactive T cell population with a peak of around 2 days post exposure [2,4]. In order to determine if the reduced proliferative responses seen with the ITK null T cells in vitro was also seen in vivo, we exposed WT and ITK null animals to SEB and determined the percentages of Vβ8 and Vβ6 positive cells in the CD4^+ ^population by flow cytometry after 2, 4 and 5 days as a measure of T cell expansion. T cells bearing Vβ8 TcR chains are responsive to SEB stimulation while those that bear Vβ6 TcR chains are not, so we analyzed these two populations of T cells. As previously reported, in WT animals, we observed an approximately 4 fold expansion in Vβ8 CD4^+ ^ T cells in spleen and lymph nodes following SEB exposure (as compared to exposure to PBS, fig 3a,b), while Vβ6 CD4^+^ T cell populations were largely unchanged (data not shown). By contrast, there was statistically significantly less expansion of ITK null Vβ8^+^CD4^+^ T cells following SEB exposure, although these cells still expanded (Fig. 3a,b) As previously reported, the percentages of T cells bearing Vβ6 did not change over this period in either the WT or ITK null mice (data not shown and [4]). Thus the absence of ITK results in reduced T cell expansion *in vivo* in response to SEB. 

### Defective SEB induced IL-2 secretion *in vivo *in ITK null mice

**Figure 4:**
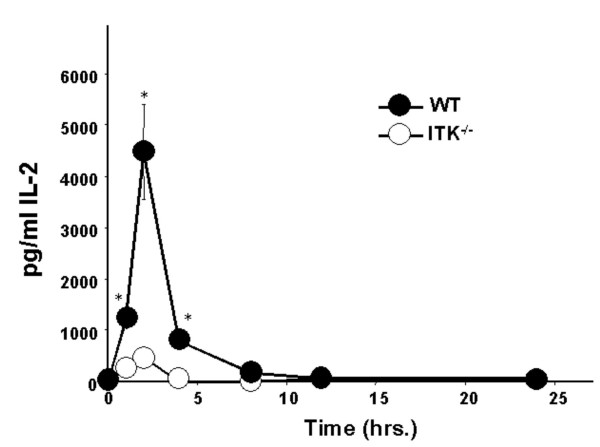
**ITK null mice secrete less IL-2 in response to SEB exposure *in vivo***. WT (filled circles) and ITK deficient (open circles) mice were injected i.v. with 50 μg SEB and blood sampled after 1, 2, 4, 8, 12 and 24 hrs. post injection. IL-2 in serum was determined by ELISA.

T cells from ITK null mice proliferate less *in vitro *and *in vivo*, and secrete significantly less IL-2 *in vitro *in response to SEB. We therefore determined if they would also secrete less IL-2 following SEB stimulation *in vivo*. Groups of mice were exposed to SEB i.v. and at various time points their serum was taken and analyzed for IL-2 production. We found that in comparison to *in vitro *IL-2 production, when WT mice were exposed to SEB *in vivo *they produced IL-2 within 1 hr., which peaked at around 2 hrs. (Fig. 4). In addition, consistent with the *in vitro *stimulation, ITK deficient mice secrete significantly less IL-2 in response to SEB *in vivo *(Fig. 4). Based on these data we conclude that *in vivo *IL-2 production occurs earlier than observed *in vitro*, and that ITK null T cells continue to exhibit defects in IL-2 production in response to SEB *in vivo*, even in the presumed presence of adequate costimulatory signals *in vivo*.

### Defective phosphorylation of c-jun induced by SEB in ITK null T cells

**Figure 5:**
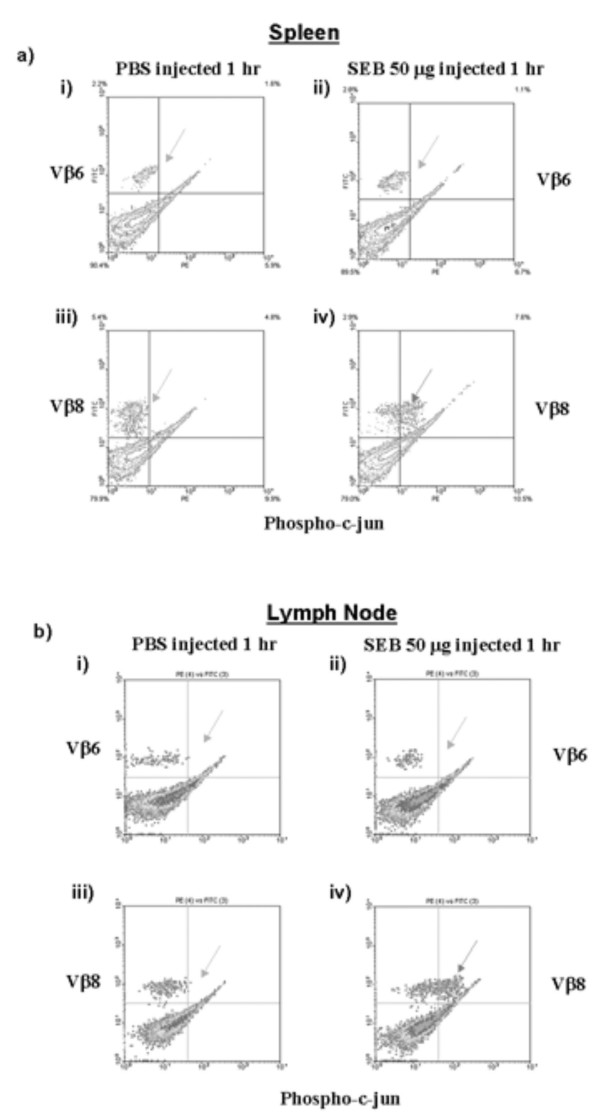
**SEB induces activation of the JNK pathway specifically in responding T cells *in vivo***. WT mice were injected with 50 μg SEB or PBS i.v. and sacrificed after 1 hr. Spleen and lymph nodes were harvested and analyzed for the presence of phosphorylated c-jun in the Vβ8^+ ^and Vβ6^+ ^T cell populations. **a) Spleen: (i) **phosphorylation of c-jun in Vβ6^+ ^T cells from PBS injected mice. **(ii) **phosphorylation of c-jun in Vβ6^+ ^T cells from SEB injected mice. **(iii) **phosphorylation of c-jun in Vβ8^+ ^T cells from the same PBS injected mice in (i). **(iv) **phosphorylation c-jun in Vβ8^+ ^T cells from the same SEB injected mice in (ii). **b) Lymph Node: (i) **phosphorylation of c-jun in Vβ6^+ ^T cells from PBS injected mice. **(ii) **phosphorylation of c-jun in Vβ6^+ ^T cells from SEB injected mice. **(iii) **phosphorylation of c-jun in Vβ8^+ ^T cells from the same PBS injected mice in (i). **(iv) **phosphorylation c-jun in Vβ8^+ ^T cells from the same SEB injected mice in (ii). Arrow points to the population of cells responding to SEB stimulation by activation of the JNK pathway by inducing c-jun phosphorylation.

The JNK MAPK pathway has been shown to be essential for IL-2 production upon stimulation of T cells by the TcR and CD28 *in vitro *[25-28]. This pathway leads to phosphorylation of c-jun and activation of an AP-1 transcription factor complex that is required for IL-2 transcription [28]. While activation of the JNK pathway leading to phosphorylation and activation of c-jun has been demonstrated in T cells following TcR and CD28 crosslinking *in vitro*, as well as by peptide antigen stimulation *in vivo*, it is not clear whether the SAG SEB activates this pathway *in vivo *[29,30]. We therefore tested whether SEB could induce phosphorylation of c-jun in WT T cells stimulated with SEB *in vivo*. To do this, we adapted a method initially used by Jenkins and colleagues to analyze phosphorylation of c-jun following *in vivo *exposure of antigen. In this protocol, mice were exposed to SEB, then cells from lymph nodes, spleen and blood rapidly isolated and fixed, and antibodies specific for phosphorylated c-jun used to analyze its phosphorylation. In addition, cells were stained with antibodies specific to Vβ8 or Vβ6 to detect SEB responsive and non-responsive T cells respectively. Flow cytometry was employed to detect phosphorylation of c-jun [30]. Figure 5 demonstrates that 1 hr. after intravenous exposure to SEB, Vβ8^+ ^SEB reactive T cells in spleen and lymph nodes contain higher levels of phosphorylated c-jun (cf. Figs. 5a, b iii & iv). Similar results were found in animals exposed to SEB i.p. (data not shown). By contrast, T cells non-reactive to SEB in the same animals, those bearing Vβ6^+ ^TcRs, did not have any increase in phosphorylated c-jun (cf. Figs. 5a, b, i & ii). This demonstrates that in the same animal, only those T cells that interact with and can be activated by SEB respond by phosphorylation of c-jun, while at the same time, those T cells that are not reactive are not activated, demonstrating specificity. Similarly, animals injected with PBS showed no such change in phosphorylated c-jun in either the SEB reactive or non-reactive T cell populations, indicating that this was an SEB mediated event (Fig. 5a–b). Other controls including secondary reagents alone demonstrate the specificity of antibody staining (data not shown).

**Figure 6:**
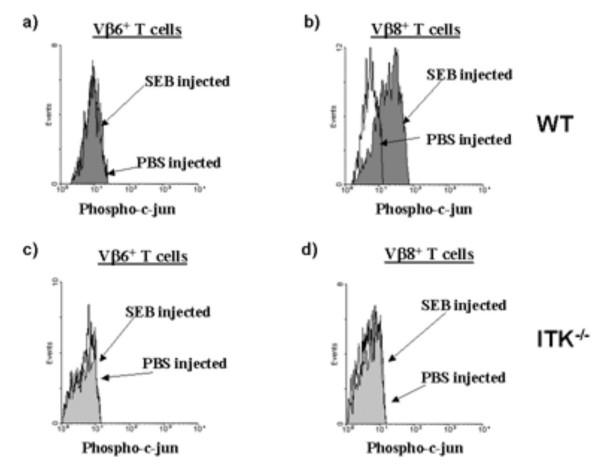
**ITK is required for SEB mediated induction of c-jun phosphorylation in responding T cells *in vivo***. WT (top panels) or ITK null (bottom panels) mice were injected with 50 μg SEB or PBS i.v. and sacrificed after 1 hr. Splenocytes were then analyzed for the presence of phosphorylated c-jun in the Vβ8^+ ^and Vβ6^+ ^T cell populations. a) Vβ6^+ ^T cells from WT mice; b) Vβ8^+ ^T cells from WT mice; c) Vβ6^+ ^T cells from ITK null mice; d) Vβ8^+ ^T cells from ITK null mice.

**Figure 7:**
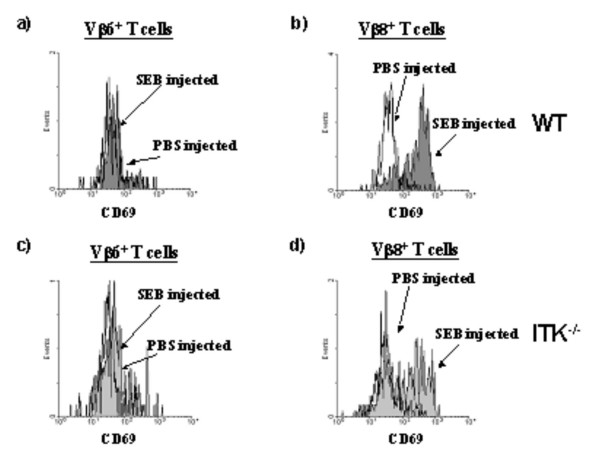
**ITK is not necessary for SEB induced upregulation of CD69 inresponding T cells *in vivo***. WT (top panels) or ITK null (bottom panels) mice were injected with 50 μg SEB or PBS i.v. and sacrificed after 2 hrs. Splenocytes were then analyzed for the presence of CD69 on the Vβ8^+ ^and Vβ6^+ ^T cell populations. a) Vβ6^+ ^T cells from WT mice; b) Vβ8^+ ^T cells from WT mice; c) Vβ6^+ ^T cells from ITK null mice; d) Vβ8^+ ^T cells from ITK null mice.

We next determined whether T cells lacking ITK could induce c-jun phosphorylation in response to SEB activation *in vivo*. ITK null mice were exposed to SEB as described above, and phosphorylation of c-jun determined in Vβ8^+ ^T cell population (SEB reactive) or Vβ6^+ ^T cell population (non-reactive) in the same animals. These experiments show that in the absence of ITK, SEB does not lead to phosphorylation of c-jun in either T cell population (Fig. 6a–d). Thus, the absence of ITK results in the lack of SEB phosphorylation of c-jun, which could affect the ability of these T cells to produce IL-2. To determine if this lack of c-jun phosphorylation in ITK null T cells in response to SEB reflected global nonresponsiveness in these cells, we determined whether these cells were indeed activated by SEB exposure. To do this, we measured increases in CD69 expression, a cell surface protein that is rapidly expressed by activated T cells in a Ras/MAPK dependent manner [31-33]. Similar experiments were performed as those examining c-jun phosphorylation, except that we used specific antibodies against CD69 to determine if the SEB reactive cells had been activated. Figure 7 demonstrates that Vβ8^+^CD4^+ ^T cells but not Vβ6^+^CD4^+ ^T cells from both WT and ITK null mice exhibited increases in CD69 expression 2 hours following SEB exposure, indicating that in both cases, the T cells were responding to SEB activation (Fig. 7). However, SEB induced CD69 expression on a higher percentage of Vβ8^+^CD4^+ ^SEB responding T cells in WT mice than in ITK null mice (73.5 +/- 12% in WT vs. 43.6 +/- 0.5% in ITK null). However, there was no difference in the MFI of CD69 expression WT responding T cells vs. ITK null responding T cells (362.5 _+/- 6.3 in WT vs. 375 +/- 11.3 in ITK null). In addition, there was no difference in the expression of CD25 on WT or ITK null T cells (data not shown). Thus the lack of SEB induced c-jun phosphorylation in ITK null T cells seems to reflect the specific role of ITK in regulating this event in response to SEB exposure in vivo.

### Similar toxicity of SEB on WT and ITK null mice

Exposure of mice to SEB and LPS results in toxicity culminating in death [1]. We therefore analyzed the effect of exposure to SEB/LPS on ITK null mice. However, in contrast to the results with IL-2 secretion and T cell expansion, both mice were affected similarly (Table 1). Thus the same proportion of mice became sick following exposure to SEB and LPS, suggesting that although the absence of ITK affects IL-2 secretion following SEB exposure, its absence does not affect the ability of SEB to induce sickness in these mice.

## Discussion

It is well established that exposure to SEB results in large scale cytokine production, which in part is responsible for symptoms of exposure to this toxin [1,2,35]. T cells have been shown to be largely responsible for this excessive cytokine production [35,36]. Current suggestions for pharmacologically blocking the symptoms of SEB exposure include T cell signal transduction inhibitors such as CsA and Perfenidone, however, these agents have significant side effects [37,38]. Here we present a promising target for inhibition IL-2 production induced by SEB exposure, the tyrosine kinase ITK. We show that mice lacking ITK have significantly reduced T cell expansion and IL-2 secretion upon exposure to SEB in vivo. Furthermore, we show that the SEB induced signaling pathway leading to c-jun phosphorylation, an indication of JNK pathway activation, is significantly reduced in ITK null T cells, although these T cells could respond to SEB activation by upregulating CD69, and there was no difference in the overall toxicity of SEB/LPS in these mice compared to WT mice.

Previous work performed *in vitro *suggests that ITK is required for the anti-TcR/CD3 antibody mediated activation of the transcription factor NFAT, via regulation of calcium influx into TcR stimulated T cells [19,39]. ITK null T cells also exhibit reduced AP-1 DNA binding activity when stimulated *in vitro *with anti-CD3 antibodies [19]. It has also been shown that JNK activation lies in part downstream of ITK following TcR crosslinking *in vitro *[23]. However, these experiments were performed *in vitro *and it is not clear that effects seen *in vitro *would reflect what happens *in vivo*, since other cell-cell interactions may allow for activation of pathways that are not seen using *in vitro *activation with antibodies. Our data however, suggest that indeed, ITK lies downstream of the TcR and is required for IL-2 secretion and c-jun phosphorylation *in vivo*. Corroborating a role for c-jun activation in IL-2 production, mice carrying a dominant negative c-jun have much reduced IL-2 secretion *in vitro *[28] (mice lacking c-jun die during embryogenesis [40]). Similarly, JNK has been implicated in IL-2 secretion by T cells *in vitro *[25-27]. Our data lend support to this idea that the JNK-c-jun pathway is involved in the production of IL-2 by T cells *in vivo*.

Curiously, the immunosuppresant CsA has been shown to inhibit the effects of SEB exposure in mice if delivered prior to SEB exposure [36], however it does not inhibit SEB induced effects in monkeys if delivered at the same time as SEB [38]. The effects of CsA on SEB induced symptoms has been suggested to be due to its effects on inhibiting IL-2 and other cytokine production due to inhibition of activation of the transcription factor NFAT [36,41], however, CsA also inhibits activation of the JNK pathway following TcR/CD3 and CD28 stimulation [29,30], and so CsA pretreatment may act to prevent early T cell activation of these pathways, thus blocking cytokine production and protecting mice from the effects of subsequent SEB exposure.

Mice lacking ITK exhibit reduced T cell responses in vitro, but also have reduced percentages of CD4^+ ^T cells (approximately 60–70% of WT, [16]). While this would be expected to reduced the overall T cell response in vivo to SEB exposure, if these cells were able to respond to SEB, we would expect an equivalent reduction in the amount of IL-2 secreted in vivo in response to SEB. We would also expect to see an equivalent reduction in proliferation in vitro. However, in vivo, we observed much reduced IL-2 secretion, and reduced expansion of Vβ8^+^CD4^+ ^T cells, although this was not as dramatic as the reduction in IL-2 secretion. Indeed, it has been observed that SEB induced T cell expansion in IL-2 null mice, indicating that IL-2 is not necessary for expansion of T cells in vivo following SEB exposure. However, even with the reduced T cell responses observed in ITK null mice, they still suffer from SEB/LPS induced toxicity similar to that seen in the WT mice. It should be noted however, that C57BL/6 mice are much less sensitive to SEB induce lethal shock than Balb/c mice [34]. Thus we observed very few deaths in our experiments, and ITK may protect Balb/c mice from actual death. We are currently crossing these mice onto the Balb/c background to determine if this is the case.

## Conclusion

In conclusion, we have show that ITK is required for IL-2 production induced by SEB *in vivo and in vitro*. We have also shown that ITK may regulate signals leading IL-2 production in part by regulating phosphorylation of c-jun. However, mice lacking ITK exhibit similar responses to SEB toxicity. The data suggest that the inability of T cell lacking ITK to produce IL-2 cannot be overcome by SAG stimulation, and that perturbing T cell activation pathways leading to IL-2 production does not necessarily lead to improved responses to SEB toxicity.

## Methods

### Mice

Wild type mice and ITK-deficient mice (C57BL/6 background, [16]) between 8 and 10 weeks were used in all experiments. RAG1^-/- ^mice were a kind gift of Dr. Eric Harvill (Penn State University) and were similarly used between 6–10 weeks of age. The Institutional Animal Care and Use Committee of The Pennsylvania State University approved all experiments.

### *In vivo *expansion assays

WT and ITK deficient mice were injected intraperitoneally (i.p.) with 50 μg SEB in PBS (Sigma-Aldrich, St. Louis, MO) and after 2 days, were sacrificed and splenocytes stained with antibodies specific for Vβ8 (SEB reactive T cells) or Vβ6 (non-reactive T cells) and CD4 directly conjugated to FITC and PE respectively (BDPharmingen, San Diego, CA). Alternatively, mice were injected i.p. with 50 μg SEB and eye-bled every 2 days for 5 days. Following lysis of red blood cells, the remaining cells were stained as described above for the splenocytes. Lymphocytes were identified by flow cytometry by their forward and side scatter characteristics.

### Intracellular staining

We used a protocol reported by Zell et al to determine the phosphorylation status of c-jun, as a measure of activation of the JNK-c-jun pathway in SEB responding T cells. WT and ITK deficient mice were injected i.p. or intravenously (i.v.) with 50 μg SEB and allowed to survive for an 1 hr. Similar results were found with both routes of administration. Animals were sacrificed and spleens, lymph nodes, and blood rapidly isolated, and dounced in 2% paraformaldehyde to rapidly fix the cells as previously described [30]. Cells were stained with antibodies to Vβ8 or Vβ6 directly conjugated to PE (BDPharmingen, San Diego, CA), then permeabilized with saponin. Cells were then stained for intracellular phosphorylated-c-jun with a monoclonal antibody against phosphorylated c-jun (IgG_1_, Cell Signaling, Beverly, MA), followed by biotinylated rabbit anti-mouse IgG_1 _and streptavidin conjugated to FITC. Controls included secondary reagents alone. Upregulation of CD69 by SEB in vivo was performed using similar approaches, except that T cells were not fixed or permeabilized prior to staining with PE-conjugated anti-CD69 monoclonal antibody (BDPharmingen, San Diego, CA). This was followed by analysis by flow cytometry, with post analysis performed using the WinMDI program 2.8 (The Scripps Research Institute, La Jolla, CA).

### *In vitro *proliferation

Lymph nodes and spleens were isolated from WT and ITK deficient mice and pooled. Red blood cells were removed using ACK lysis buffer, and cells resuspended in complete RPMI. Cells were then left unstimulated or stimulated with varying concentrations of SEB (5 μg/ml, 0.5 μg/ml, 0.05 μg/ml, 0.005 μg/ml, and 0.0005 μg/ml) at a concentration of 2 × 10^6 ^cells/ml. These cells were incubated at 37°C for 5 days and pulsed once a day for 5 days with 0.5 μCi tritiated thymidine per well for 12 hrs. before harvest. T cells were purified from WT and ITK null mice using MiniMACS separation columns (Miltenyi Biotec Inc, Auburn, CA), and plated at 2 × 10^6 ^cells/ml and stimulated as above, in the presence of splenocytes from RAG null mice pre-treated with mitomycin C. All experiments were done in triplicate.

### *In vitro *cytokine analysis

Pooled lymphocytes and splenocytes from WT and ITK deficient mice were incubated with varying concentrations of SEB (5 μg/ml, 0.5 μg/ml, 0.05 μg/ml, 0.005 μg/ml, and 0.0005 μg/ml) for 5 days as described above for *in vitro *proliferation. Supernatants were sampled once a day over this period and analyzed for IL-2 using ELISAs according to the manufacturer's recommendations (BDPharmingen, San Diego, CA).

### *In vivo *cytokine analysis

WT and ITK deficient mice were injected with 50 μg SEB i.v., and serum isolated from cardiac blood from mice 1, 2, 4, 8, 12, and 24 hrs. post SEB exposure and used to perform an ELISA to determine IL-2 levels. PBS injected mice served as controls, and there was no change in IL-2 secretion in these animals.

### Statistics

Values were compared using student's t test and considered significant if p < 0.05.

## List of abbreviations used

ITK Interleukin-2 inducible T cell Kinase

IL-2 Interleukin-2

SEB Staphylococcal Enterotoxin B

SAG Superantigen

Th1 T-helper 1 cells

Th2 T-helper 2 cells

Syk Spleen tyrosine kinase

Zap-70 Zeta chain Associated protein-70

Lck Lstra cell kinase

MAPK Mitogen Activated Protein Kinase

ERK Extracellular signal Regulated Kinase

JNK c-jun N-terminal kinase

NK Natural Killer cells

LCM Lymphocytic Choriomeningitis

VS Vesicular Stomatitis

PMA Phorbol Myristic Acid

PBS Phosphate Buffered Saline

NFAT Nuclear Factor of Activated T cells

AP-1 Activator protein 1

CsA Cyclosporin A

## Authors' contributions

AA designed the experiments. MJR and JH performed experiments leading to figure 1. MJR performed experiments leading to figures 2, 3, 4, 5, 6, 7. AA and AJH provided financial support and supervision. MJR, AJH and AA wrote the manuscript. All authors read and approved the final manuscript.

## Figures and Tables

**Table 1:** Effect of SEB on health and survival of WT and ITK null mice. Mice (WT or ITK null) were injected with the indicated amount of LPS, followed 4 hours later by 50 μg SEB, both delivered Ip. Alternatively, mice were injected with 20 mg. D-Gal and 50 μg SEB at the same time. All injections were delivered intraperitoneally. Mice were then monitored for the presence of ruffled fur, mucous in feces and lethargy. *mice had ruffled fur, mucous in feces and were lethargic. **mouse died over the course of the experiment. ***mouse had to be euthanized due to severity of sickness.

**Mice**	**Challenge**	# Sick/n*	**# Dead/n**
WT	50 μg SEB + 150 μg LPS	14/15	1/15**
ITK^-/-^	50 μg SEB + 150 μg LPS	11/12	1/12***
WT	50 μg SEB + 20 mg D-Gal	10/11	1/11**
ITK^-/-^	50 μg SEB + 20 mg D-Gal	11/11	0/11
